# Intracranial Vasospasm After Evacuation of Acute Spontaneous Subdural Hematoma

**DOI:** 10.7759/cureus.15284

**Published:** 2021-05-27

**Authors:** Andrew J Witten, Josue D Ordaz, Vincent J Alentado, Bradley Bohnstedt

**Affiliations:** 1 Neurological Surgery, Indiana University School of Medicine, Indianapolis, USA; 2 Neurological Surgery, Indiana University, Indianapolis, USA

**Keywords:** spontaneous subdural hematoma, acute subdural hematoma, intracranial vasospasm, subdural hematoma evacuation, delayed ischemic neurological deficit

## Abstract

Cerebral vasospasm is a well-known entity following aneurysmal subarachnoid hemorrhage. While it has been described in trauma, it has been much less studied. There have been no previous reports of cerebral vasospasm following spontaneous subdural hematoma or after subdural hematoma evacuation. In this case report, we present a 38-year-old otherwise healthy female who suffered an acute spontaneous subdural hematoma. After surgical evacuation of her hematoma, she developed neurologic decline. Computer tomography angiography demonstrated intracranial vasospasm. She was treated with blood pressure augmentation and nimodipine. She went on to make a full neurologic recovery.To our knowledge, this is the first reported case of cerebral vasospasm after acute spontaneous subdural hematoma or after subdural hematoma evacuation, and the patient recovered without sequelae. The promising outcome of this case may provide a framework for future similar cases. Neurosurgeons and intensivists should keep cerebral vasospasm in their differentials for patients who have neurologic decline after craniotomy for acute subdural hematoma and have an otherwise negative scan for new acute abnormality.

## Introduction

Cerebral vasospasm is a well-described phenomenon that generally occurs after aneurysmal subarachnoid hemorrhage (SAH). However, any clinical scenario that brings blood into the cerebral subarachnoid space, such as non-aneurysmal, post-surgical, and traumatic SAH, can cause the delayed, secondary consequence of intracranial vasospasm [1,2]. Delayed cerebral ischemia is defined as the development of new focal neurological signs and/or deterioration in the level of consciousness caused by conditions such as acute vasospasm where there is a sudden contraction of muscular arterial walls. The pathophysiology of vasospasm is poorly understood, but rapid diagnosis is critical to initiate appropriate therapy and prevent or mitigate cerebral ischemia that, if left unchecked, can cause stroke and lead to neurological deterioration and sequelae.

Acute spontaneous subdural hematomas (aSDHs) are defined as non-traumatic acute subdural bleeding from the arterial origin, without any traumatic history or vascular anomaly. They are extremely rare, making up 2% to 6.7% of all acute subdural hematomas (SDH) [3-5]. To our knowledge aSDHs have not been reported to be associated with delayed cerebral vasospasm [6]. We present a case of post-aSDH evacuation complicated by cerebral vasospasm. We hope this case will help broaden the differential diagnoses of neurological deterioration after SDH evacuation and give an outline of a successful treatment algorithm.

## Case presentation

Pre-operative course

**Figure 1:**
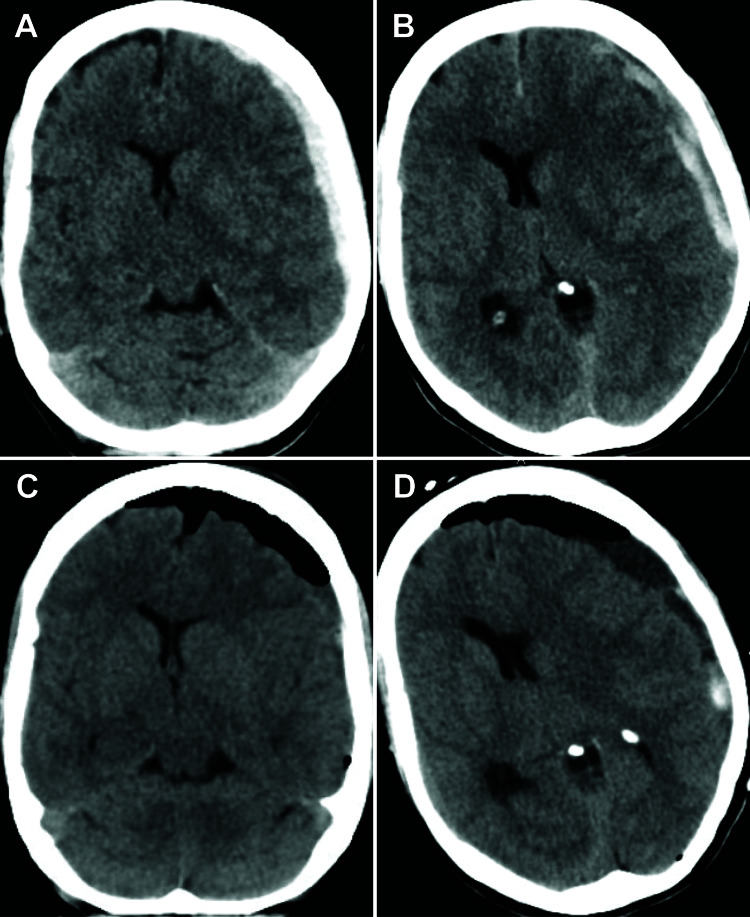
Spontaneous acute subdural hematoma. (A) Subdural hematoma at initial hospital presentation. (B) Interval increase of subdural hematoma four days after initial presentation. (C) One-day post-operative head CT demonstrating improved midline shift. (D) CT head on the post-operative day 2 demonstrating increased midline shift but without significant acute blood or other compressive etiology.

The patient is a 38-year-old healthy female who presented to the emergency department with a spontaneous headache and expressive aphasia. CT head (CTH) demonstrated a 7-mm acute SDH (Figure 1A) with 2 mm midline shift. She was adamant about her lack of trauma history, was alert and oriented, and did not report any sort of memory loss. Detailed physical examination did not reveal any signs of trauma. Due to the lack of trauma history, a CT angiogram (CTA) and cerebral angiogram were obtained to rule out an underlying vascular lesion. These tests were negative for pathology. Repeat CTH showed stability of the hematoma. She subsequently left the hospital against medical advice on hospital day 3.

Four days after initial discharge, the patient presented with increased headaches, vomiting and lethargy. She had no focal neurologic deficit. CTH showed interval increased mixed density SDH measuring 1.1 cm with 8 mm of midline shift (Figure 1B). The patient was taken to the operating room for a small frontoparietal craniotomy for evacuation.

Post-operative course

**Figure 2:**
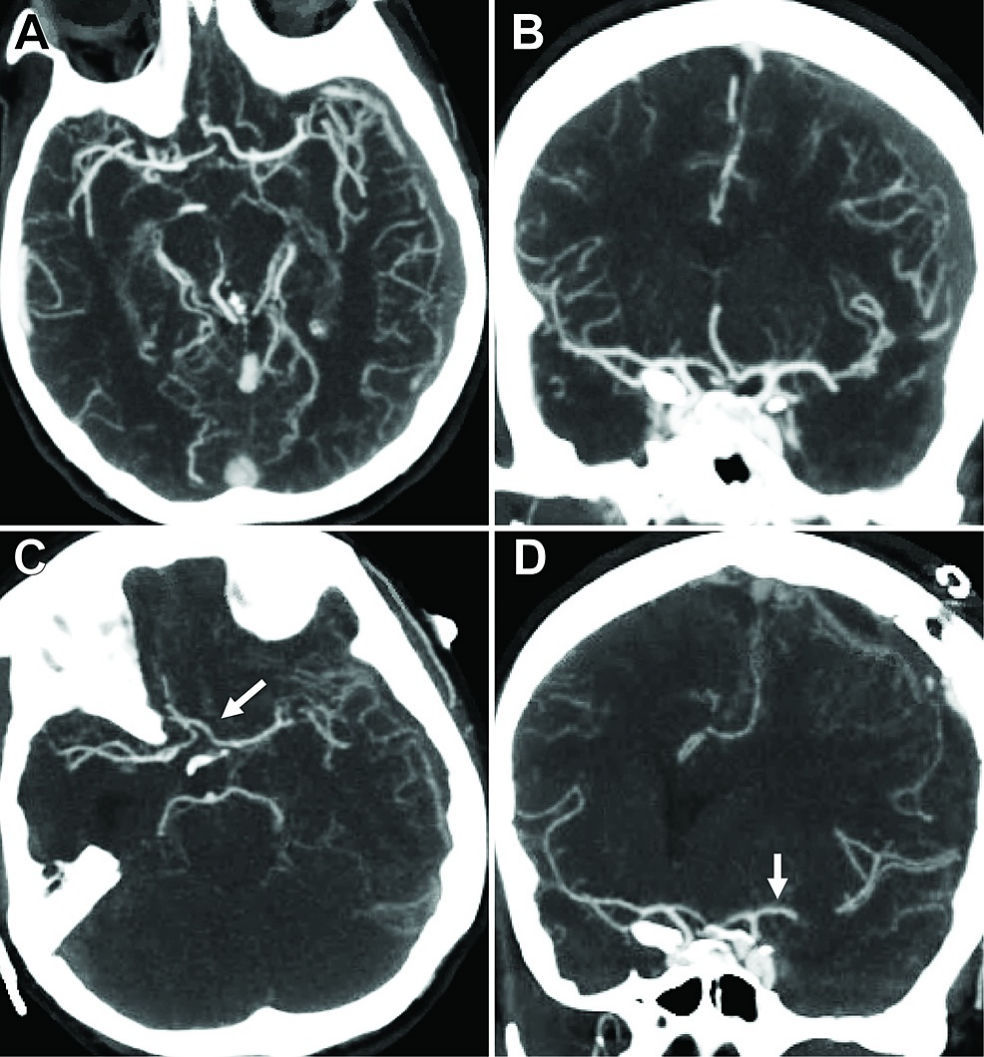
Development of cerebral vasospasm after subdural hematoma evacuation. (A, B) Axial and coronal views, respectively, demonstrating preoperative cerebral vasculature. Vessels appear normal caliber. (C, D) Vasospasm is noted in the L>R supraclinoid and L anterior cerebral and middle cerebral arteries when compared to the preoperative CT angiogram (white arrows). The cortical branches also have notably less filling.

The patient returned to the ICU post-operatively. On post-operative day (POD) 1, CTH demonstrated improved midline shift (Figure 1C) and the patient returned to her neurological baseline. On POD 2, the patient developed increased confusion, restlessness and a new left-sided facial droop. CTH demonstrated increased midline shift, but no compressive etiology (Figure 1D). The patient was placed on neurotelemetry, started on supplemental oxygen, and her head of bed was placed flat. A CTA was performed, which demonstrated L>R supraclinoid, L M1/M2 and L A1-A3 vasospasm compared to the preoperative CTA (Figures 2A-2D). Blood pressure was augmented with norepinephrine for a systolic blood pressure goal of >160 mmHg. Fluids were started at 100 mL/hr. There was significant clinical improvement in exam within an hour of blood pressure augmentation, verifying our suspicion of vasospasm as the main cause of her symptoms. The patient was also started on nimodipine to prevent delayed ischemic neurological deficit (DIND). Transcranial Dopplers were obtained for the following four days. Mean flow velocities did not reach vasospasm levels. Patient improved and was subsequently discharged home on POD 6. At her one-month clinic follow-up appointment, the patient was neurologically intact and only complaining of incisional pain. CTH demonstrated significant improvement in SDH.

## Discussion

Cerebral vasospasm can lead to DINDs. Approximately half of the cases of intracranial vasospasm develop delayed cerebral ischemia and 20% of those cases result in significant morbidity and/or mortality [2]. The normal timeline for intracranial vasospasm after SAH is typically 4-14 days [2]. However, an earlier onset often occurs for cerebral vasospasm caused by TBI; typically, before day 3 [2,7]. Duration of symptoms is also shorter for post-traumatic vasospasm in the absence of SAH, with most cases lasting about 1.25 days [8]. Nine days following the aSDH, our patient had neurological decline caused by intracranial vasospasm as confirmed with CTA. Without visible SAH on MRI or CT and the time course following surgery, we speculate that the SDH evacuation played an essential role in the development of intracranial vasospasm. To our knowledge, the atraumatic origin of SDH and vasospasm following evacuation has never been reported in the literature.

Various theories have been proposed as possible mechanisms for intracranial vasospasm, but the mechanisms are still incompletely understood. One possible method is the contraction of cerebral arterial smooth muscle cells and impairment of vasodilatory activity due to prostacyclin/thromboxane A2 imbalance. Another theory is hemoglobin degradation where oxyhemoglobin induces inhibition of acetylcholine-mediated vasodilation. Finally, inflammatory cascade and immunoreactive processes appear to be possible contributors to vasospasm [2,9].

If recognized early, the outcome of intracranial vasospasm is favorable. Formal cerebral angiography remains the gold standard for the evaluation of vasospasm [10]. CTA remains a useful tool to diagnose cerebral vasospasm. However, without a baseline study, it can be difficult to diagnose and render the study non-specific. In our case, a pre-operative CTA provided a valuable comparison to the CTA after neurological decline to solidify an accurate diagnosis of cerebral vasospasm. Compared to her pre-operative CTA, we were able to observe significant narrowing of supraclinoid internal carotid, anterior cerebral, and middle cerebral arteries. The theories presented previously are likely the same mechanism of action for the post-hematoma evacuation vasospasm, although subarachnoid blood was not present on CT.

When symptomatic vasospasm is detected, emergent cerebral perfusion augmentation and neuroprotection is required to prevent DIND. We increased the parameter to >160 mmHg to improve perfusion and observed almost immediate improvement of symptoms. Calcium channel antagonists have traditionally been used because of its neuroprotective properties caused by arterial smooth muscle cell relaxation [11]. It is the only FDA-approved medical therapy for the treatment of cerebral vasospasm [12]. Our patient’s vasospasm appeared to resolve after initiation of nimodipine and systolic blood pressure increase, and this was confirmed with transcranial Doppler monitoring for four days.

## Conclusions

This case report shows that cerebral vasospasm is not exclusive to SAH and can be seen in SDH following hematoma evacuation. Clinicians must remain on high alert for this treatable entity after a neurologic decline following a traumatic hemorrhage and/or craniotomy. It is important that vasospasm be detected early and emergent cerebral perfusion augmentation and neuroprotection be initiated to prevent DIND. Although sometimes unavailable, pre-operative CTAs provide a valuable comparison to the CTA after a neurological decline to solidify an accurate diagnosis of cerebral vasospasm. After this report, it is possible that more cases of cerebral vasospasm associated with post-hematoma evacuation will be identified and reported. Vasospasm should be included in the differential diagnoses for post-operative neurological decline after SDH evacuation.
